# Implications of BRAF V600E mutation in gliomas: Molecular considerations, prognostic value and treatment evolution

**DOI:** 10.3389/fonc.2022.1067252

**Published:** 2023-01-04

**Authors:** Vincenzo Di Nunno, Lidia Gatto, Alicia Tosoni, Stefania Bartolini, Enrico Franceschi

**Affiliations:** ^1^ Department of Oncology, AUSL Bologna, Bologna, Italy; ^2^ Nervous System Medical Oncology Department, IRCCS Istituto delle Scienze Neurologiche di Bologna, Bologna, Italy

**Keywords:** BRAF, glioma, glioblastoma, dabrafenib, trametinib, vemurafenib, encorafenib, MAPK-MEK

## Abstract

Gliomas are molecularly heterogeneous brain tumors responsible for the most years of life lost by any cancer. High-grade gliomas have a poor prognosis and despite multimodal treatment including surgery, radiotherapy, and chemotherapy, exhibit a high recurrence rate. There is a need for new therapeutic approaches based on precision medicine informed by biomarker assessment and BRAF, a key regulator of MAPK signaling pathway, influencing cell differentiation, proliferation, migration and pro-tumorigenic activity, is emerging as a promising molecular target. V600E, is the most frequent BRAF alteration in gliomas, especially in pediatric low-grade astrocytomas, pleomorphic xanthoastrocytoma, papillary craniopharyngioma, epithelioid glioblastoma and ganglioglioma. The possible application of BRAF-targeted therapy in gliomas is continuously growing and there is preliminary evidence of prolonged disease control obtained by BRAF inhibitors in tumors harboring BRAF V600E mutation. The possibility of introducing targeted therapies into the treatment algorithm represents a paradigm shift for patients with BRAF V600E mutant recurrent high-grade and low-grade glioma and BRAF routine testing should be considered in clinical practice. The focus of this review is to summarize the molecular landscape of BRAF across glioma subtypes and the novel therapeutic strategies for BRAF V600E mutated tumors.

## Introduction

The V-RAF murine viral oncogene homolog B1 (BRAF) is a RAF1 serine/threonine protein kinases member which is implicated as an oncogene in several malignancies including central nervous system (CNS) primary tumors (1–4). Physiologically, BRAF is activated by RAS (Rat Sarcoma virus) proteins which are small GTPase proteins (1–4). Once activated, BRAF homo or heterodimerize activating the mitogen-activated protein kinases MEK1 and MEK2 by phosphorylation. Of note, MEK 1 and MEK2 are encoded by the two genes: mitogen-activated protein kinase 1 and 2 known as MAPK1 and 2 respectively (1–4). Finally, the two Extracellular Signal-regulated kinases (ERK) ERK 1 and ERK2 activated by MEK1/2 stimulate cell survival, proliferation, and dedifferentiation modulating transcriptional activity in the nucleus (1–4). The BRAF activity can be pathologically switched on the active open configuration due to several causes including BRAF gene mutations (point mutations, fusion, or in-frame deletion) as well as mutations occurring on regulatory genes preventing BRAF activation.

**Figure 1:**
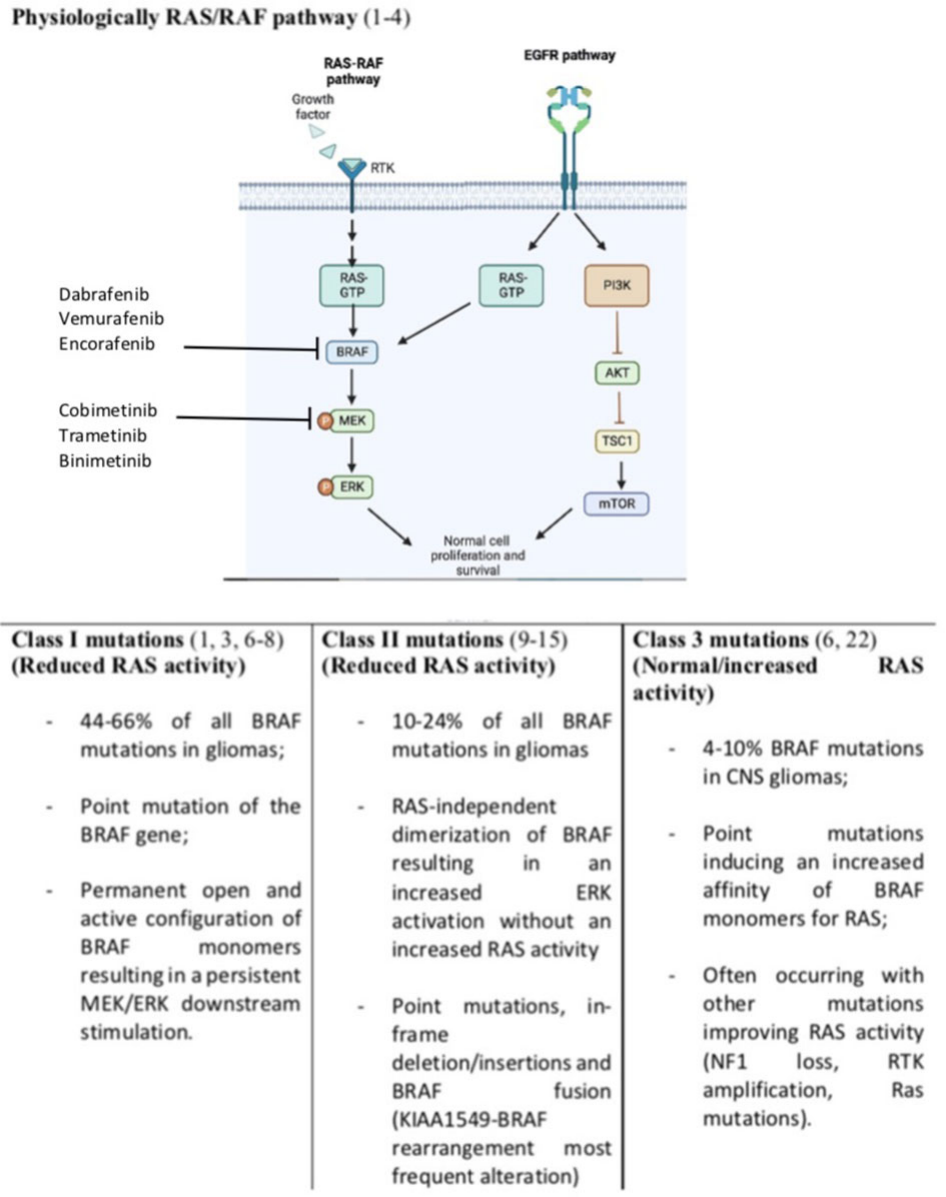
Normal RAS/RAF pathway and BRAF mutations resulting in survival and growth stimulation. Incidence of BRAF mutations are referred to glioma tumors only. We included also EGFR (Epidermal Growth Factor Receptor) pathway and its connection with RAS stimulation. Notably EGFR activation lead also to Phosphatidyl-Inositol 3-Kinase (PI3K), inhibition of the protein kinase B (Akt) and activation of the mechanistic target of rapamycin (mTOR) converging on cell survival and progression.

Overall about 100 mutations of the BRAF gene have been identified in cancers and grouped into three different classes (5). By the use of cBioPortal Schreck KC et al. were able to identify several kinds of BRAF mutations occurring in glioma and belonging to all three classes of BRAF alterations (**Figure 1**) (4). Notably, this same study reported that about 20% of the mutations detected presented an unknown or insignificant clinical significance (4).

Class I mutations constitute 44% of all BRAF mutations in CNS tumors and are associated with a point mutation of the BRAF gene. The most frequent mutation is the c.1799T>A leading to a substitution from valine to glutamic acid at position 600 (V600E) (1, 3, 6–8). No data about BRAFV600K mutation have been reported on patients with gliomas. Valine can be substituted for other amino acids. However, the V600E mutation is the only alteration of this class detected on glioma. This point mutation leads to a permanent open and active configuration of BRAF monomers resulting in a persistent MEK/ERK downstream stimulation (1, 3, 6–8). This class of mutation lead to a RAS-independent activity of the BRAF monomers (1, 3, 6–8).

Class II are less frequent than class I mutations and involve several non-V600E BRAF mutations, in-frame deletions, and fusions. These mutations lead to a RAS-independent dimerization of BRAF resulting in an increased ERK activation without an increased RAS activity (9). In-frame deletions can reduce the αC helix by removing part of the β3-αC loop resulting in a BRAF monomer refractory to autoinhibition and with an increased kinases activity (10–12). Similarly, point mutations such as K601E/N/T, L597Q/V, and G469A/V/R can lead to the same BRAF constitutive activation. Also, fusions leading to RAS-independent dimerization belong to class II mutations. The most known fusion is between BRAF and KIAA1549 (13, 14). Fusions are common in low-grade gliomas and lead to the fusion of the C-terminal kinase BRAF domain to the N terminal domain of another gene (like KIAA). This led to an increased BRAF activity which is independent of upstream regulation due to the loss of the BRAF regulatory domain (13, 14). Several fusion genes have been described in glioma including BCAS1 (Brain Enriched Myelin Associated Protein 1), CCDC6 (Coiled-Coil Domain Containing 6), CDC42BPB (CDC42 binding protein kinase beta), FAM131B (Family With Sequence Similarity 131 Member B), FXR1 (FMR1 Autosomal Homolog 1), GIT2, KLHL7 (Kelch Like Family Member 7), RNF130 (Ring Finger Protein 130) and TEMEM106B (Transmembrane Protein 106B) (15–21).

Class III mutations are rarer than class I and II ones (4-10% of BRAF mutation in glioma) and contrarily to the other classes are RAS-dependent (4). Indeed, differently from other classes, these BRAF point mutations (G466E/A/V, G596D/R, and D594G) are associated to an increased affinity and response to RAS activation compared to wild-type BRAF monomers (22). Not surprisingly, these alterations often occur with other mutations which are associated with an increased RAS activity (RAS mutation, NF1 loss, RTK amplification/mutations) (6, 22).

## BRAF alterations in primary CNS tumors

BRAF alterations have been largely described in both pediatric and adult primary CNS tumors (**Table 1**). The incidence of BRAF mutations diverges according to tumor histology ranging from 1-2% and 2-5% in glioblastoma and astrocytoma to 81-95% in papillary craniopharyngioma (41).

**Table 1:** Incidence and subtype of BRAF mutations within central nervous system primary tumors in pediatric and adult population.

BRAF mutations incidence		
Pediatric tumors	BRAF alteration	Percentage and references
Pilocytic astrocytoma	BRAF-KIAA1549	70% (23, 24)
	BRAF V600E	10-12% (25)
Diffuse leptomeningeal glioneuronal tumor	KIAA1549-BRAF	70-75% (26, 27)
Ganglioglioma	BRAF V600E	20-60% (17, 28)
Desmoplastic infantile ganglioglioma	BRAF V600E	45% (15, 29–31)
Dysembryoplastic neuroepithelial tumor	BRAF V600E	30-80% (15, 29–31)
Adult tumors		
Glioblastoma	BRAF V600E	1-2% (32–34)
Low grade gliomas	BRAF V600E and BRAF-KIAA1549	2-5% (32, 34)
Pleomorphic xanthoastrocytoma	BRAF V600E	60% (35, 36)
Astroblastoma	BRAF V600E	38% (37)
Papillary craniopharyngioma	BRAF V600E	80-95% (38–40)

## Pediatric CNS tumors

In children, BRAF alterations are mainly observed in low-grade gliomas including pilocytic astrocytoma and glial-neuronal tumors (41).

The pilocytic astrocytoma is a common astrocytic tumor in children and is recognized as a WHO (world health organization) grade 1 tumor according to the CNS WHO 2021 classification (42). In these tumors, the most frequent alteration is the duplication of the chromosome 7p34 resulting in a fusion gene involving the BRAF kinase domain and the N-terminal KIAA1549 protein (up to 70% of cases) (23, 24). Even if fusions are the most frequent event, BRAF point mutation including V600E can be detected in 5-8% of cases and are mutually exclusive with KIAA1549-BRAF fusion (25).

The diffuse leptomeningeal glioneuronal tumors are glioneuronal tumors recognized by the 2021 WHO classification. These tumors spread within leptomeninges and can be sometimes misdiagnosed as meningitis. Also, these tumors show in up to 75% of cases a KIAA1549-BRAF rearrangement but differently from pilocytic astrocytoma is associated also with 1p19q codeletion (26, 27).

Within glioneuronal tumors, ganglioglioma is another well-differentiated malignancy frequently located on the temporal lobe and associated with early epilepsy onset. Within these tumors is commonly observed the BRAF V600E mutation (20-60%) and also in-frame insertion in the β3-αC loop have been described (17, 28). Other pediatric CNS tumors often reporting BRAF V600E mutations are the desmoplastic infantile ganglioglioma (45%) and the dysembryoplastic neuroepithelial tumor (30-80%) (15, 29–31).

## Adult CNS tumors

Glioblastoma (GBM) is the most frequent malignant primary CNS in adults. Despite this, BRAF V600E mutations are extremely rare and detectable in only 1-2% of cases (32–34). BRAF V600E mutated GBM presents its clinical-pathological features including epithelioid features of the tumor cell. There are still little data investigating the prognostic role of BRAF V600E mutation in GBM (32–34). BRAF V600E and canonical Isocitrate dehydrogenase (IDH) gene mutations are mutually exclusive. Nonetheless, BRAF V600E mutation can be detected with non-canonical IDH mutations (43–46).

In diffuse low-grade gliomas, BRAFV600E mutations can be found in 2-5% of cases while also the KIAA1549-BRAF rearrangements have been described (32, 34). Notably, low-grade glioma with BRAF aberrations often arises in the cerebellum (32, 34). Finally, BRAF gains are common in diffuse oligodendroglioma harboring 1p/19q loss (47).

The pleomorphic xanthoastrocytoma is a circumscribed glial tumor recognized by the WHO 2021 classification and diagnosed in pediatric patients and young adults (35, 36). It is generally associated with a favorable prognosis nonetheless, some rare cases can dedifferentiate toward an anaplastic variant characterized by an increased recurrence rate and more aggressive clinical features (35, 36). BRAF V600E mutations are commonly found in these tumors reaching an overall incidence of 60%. However, these same mutations are rare in anaplastic pleomorphic xanthoastrocytoma being detectable in only 10-12% of cases (35, 36).

Astroblastoma is a rare tumor diagnosed mainly in young adults. This tumor is characterized by the MN1 gene rearrangements (MN1 Proto-Oncogene, Transcriptional Regulator) and shows a BRAF V600E mutation in up to 38% of cases (37).

Finally, the papillary craniopharyngioma is a benign tumor of the sellar region deriving from the Rathke pouch and diagnosed exclusively in adults (38–40). These tumors have a very large incidence of BRAF V600E mutations (80-95%) and are a distinct entity compared to adamantionous Wnt-associated craniopharyngioma diagnosed in pediatric patients (38–40).

## BRAF/MEK targeted therapy in gliomas

The use of orally bioavailable small molecules BRAF inhibitors has gained significant attention in oncology after the success of FDA-approved drugs targeting the BRAF V600E mutation in melanoma, papillary thyroid cancer, BRAF mutated non–small cell lung cancer and hairy cell leukemia (48–51).

One of the most important issue to consider testing a novel drug in patients with glioma is ability of the agent to pass the blood-brain barrier and achieve therapeutic concentration.

To date, we have no data about the effective concentration reached by BRAF and MEK inhibitors in glioma patients. Nonetheless, important studies aimed to investigate the effective penetration of these agents have been carried out on melanoma patients in both clinical and pre-clinical settings. Dabrafenib, vemurafenib and encorafenib showed clinical efficacy on melanoma brain metastases (52–55). Despite this acquired clinical it has been demonstrated that all these agents reach a sub-optimal concentration explaining why brain is often the primary site of melanoma progression in course of target inhibitions (52–55). Similarly other factors could influence penetration of MEK inhibitors on the brain (56).

Despite the relatively low incidence of BRAF V600E mutations in high grade gliomas, mounting evidence has suggested that BRAF targeted therapy represents a promising treatment option for adults with BRAF mutated high grade gliomas or GBM.

Several cases of impressive response to BRAF inhibitors vemurafenib and dabrafenib in high grade gliomas have been reported, including cases of complete radiological disease and prolonged disease control (**Table 2**) (57–67).

**Table 2:** Available clinical data on BRAF V600E mutated GBM and eGBM treated with BRAF inhibitors alone or in combination.

Author	Drug	Study type	Tumor type	N. patients	Best Response
Robinson et al. (57)	vemurafenib	case report	V600E GBM	1	CR
Burger et al. (53)	dabrafenib	case report	V600E GBM	1	CR
Ceccon et al. (54)	dabrafenib	case report	V600E eGBM	1	SD
Qin C et al. (58)	dabrafenib	case report	V600E GBM	1	PR
Beba et al. (52)	vemurafenib	case report	V600E GBM	1	SD
Schreck et al. (59)	dabrafenib + trametinib	case report	V600E eGBM	1	SD
Kushnirsky et al. (60)	dabrafenib + trametinib	case report	V600E GBM	1	CR
Kanemaru et al. (61)	dabrafenib + trametinib	case report	V600E eGBM	1	PR
Johanns et al. (55)	dabrafenib + trametinib	case report	V600E eGBM	1	PR
Kaley et al. (56)	vemurafenib	basket trial	V600E GBM	11	ORR 11%
Wen et al. (62)	dabrafenib + trametinib	basket trial	V600E GBM	31	ORR 33%

Robinson et al. in 2014 described a dramatic response in a pediatric case of relapsed BRAF V600E-mutated GBM treated with vemurafenib. The patient exhibited a complete clinical and radiological response after 4 months of treatment, sustained for 6 months (65).

Interestingly, Burger et al. (60) reported three cases of recurrent malignant gliomas harboring a BRAF V600E mutation, showing a complete or nearly complete response after receiving dabrafenib as a single agent. Notably, all patients presented with markedly disseminated leptomeningeal disease at recurrence, thus their estimated life expectancy was a few weeks. All three patients achieved a complete or nearly complete response with dabrafenib and one patient obtained a dramatic radiologic and clinical response after only one week of treatment.

Higher-quality evidence on the therapeutic efficacy of BRAF inhibitors in GBM has been documented by the VE-BASKET trial (59), a basket trial of BRAF V600E mutation-positive solid tumors, evaluating the safety and effectiveness of vemurafenib 960 mg twice per day continuously after tumor progression with standard therapy. The study divided patients into 7 diverse cohorts by histology and included 24 patients with different mutant gliomas of any grade, including 11 malignant diffuse gliomas (6 GBMs and 5 five anaplastic astrocytomas). Among the whole glioma cohort, objective response rate was 25%, overall median PFS resulted 5.5 months (95% CI, 3.7 to 9.6 months) and overall median OS was 28.2 months (95% CI, 9.6 to 40.1 months). Regarding the malignant diffuse glioma subgroup, ORR was 9.1%, median PFS 5.3 months and median OS 11.9 months. The best response included one partial response and five disease stabilizations, two of them lasting more than 12 months. The results of this study were extremely heterogeneous, qualitatively depending by grading and histologic subtype. Obviously, considering the limits of repeating a brain biopsy, patients were not tested for the V600E BRAF mutations immediately before trial enrollment. Thus, when evaluating the results of the study it is necessary to consider the intratumoral heterogeneity and the possibility that in the context of the tumor mass there could be subclones lacking the BRAF mutation or resistant subclones (68).

Although initial sensitivity to RAF inhibitors and encouraging tumor responses, several mechanisms of resistance to RAF inhibitors frequently emerges. The multidrug combination strategy using BRAF and MEK inhibitors represents also an established therapeutic option in patients with BRAF mutated solid cancers, demonstrating reduced resistance without increased toxicity.

In melanoma experiences, while administering an anti-MEK agent after BRAF inhibition failure is not very effective, the administration of both drugs simultaneously improves objective responses and survival and actually represents a standard of care (69). Studies using BRAF V600E mutated high-grade glioma cells and flank xenografts demonstrated that BRAF V600E inhibitor Vemurafenib did not eliminate all tumor cells equally. A subpopulation of cells marked by CD13 expression, high asymmetric cell division, slow proliferation rates compared with CD133-negative tumor cells, is less sensitive to the anti-proliferative effects of Vemurafenib, and accumulates with treatment, suggesting a role for these cells in tumor escape from BRAF V600E inhibition and recurrence (69).

Currently, there are 3 FDA approved combinations of RAF/MEK inhibitors: dabrafenib plus trametinib, vemurafenib plus cobimetinib, and encorafenib plus binimetinib.

Preclinical studies using an orthotopic murine BRAF V600E mutated glioma model confirmed that BRAF V600E inhibitor monotherapy with dabrafenib inhibited MAPK signaling only transiently. The combination with MEK inhibitor trametinib provided a more durable suppression of the MAPK pathway, which translated to effective suppression of *in vivo* tumor growth and resulted in a significant survival benefit of the combination treatment over control and monotherapy (70).

Kushnirsky et al. (63) reported a case of complete response with the combination of dabrafenib and trametinib after the emergence of resistance to single-agent BRAF inhibitor in a patient diagnosed with MGMT unmethylated, IDH wild-type GBM harboring V600E BRAF mutation and CDKN2A/B loss. The patient, 44 years old, diagnosed with recurrent WHO grade 4 GBM, after progression to standard radio-chemotherapy, was enrolled into a clinical trial of BRAF inhibitor PLX8394 in combination with cobicistat, achieving radiographic partial response maintained for 7 months. Subsequently, he underwent multifocal radiographic progression and was treated with dabrafenib and trametinib, achieving, after 11 months of treatment, complete radiological response and complete resolution of symptoms, without significant toxicities.

Similarly, Kanemaru et al. (62) reported an impressive response to combination therapy with BRAF and MEK inhibitor in a case of BRAF V600E-mutant epithelioid GBM (eGBM) with diffuse metastatic dissemination in the spine, a condition usually associated with a poor prognosis, with a median survival of 3 months. Other reports confirmed rapid clinical and radiographical responses of dabrafenib plus trametinib in adults with high-grade gliomas, even in the setting of newly diagnosed disease, suggesting that dual inhibition is safe and effective in this population (57, 66).

Wen et al. have reported interim results from the Rare Oncology Agnostic Research (ROAR) study, a phase II, open-label, single-arm, multi-center basket trial investigating the role of the combination of dabrafenib and trametinib in BRAF mutant solid tumors (67, 71). Between 2014 and 2018, 45 patients (31 diagnosed with GBM) were enrolled into the high-grade glioma (HGG) cohort and 13 patients were enrolled into the low-grade glioma (LGG) cohort. The primary endpoint was the objective response rate (ORR) by Response Assessment in Neuro-Oncology (RANO) criteria. In the HGG cohort, 15 of 45 patients (33%) had an objective response including 3 complete responses and 12 partial responses. In the LGG cohort, 9 of 13 patients (69%) had an objective response including 1 complete response, 6 partial responses, and 2 minor responses. Grade 3 or worse adverse events were reported in 31 (53%) patients, the most common being fatigue, decreased neutrophil count and headache.

The combination dabrafenib and trametinib has also been explored in the NCT02684058 phase II study analyzing the setting of systemic first line therapy in BRAF V600E positive pediatric LGGs, with encouraging results both in terms of effectiveness and safety. 110 pediatric patients were randomized to receive dabrafenib + trametinib once daily or standard chemotherapy with Lomustine and Vincristine (72). The primary endpoint of the study was ORR, that resulted 47% with dabrafenib + trametinib arm *vs* 11% with chemotherapy arm (p <0.001). Median PFS resulted 20.1 months with dabrafenib and trametinib *vs* 7.4 months with chemotherapy (p<0.001) (72). In addition, grade ≥3 adverse events were lower in the dabrafenib and trametinib arm (47% *vs* 94%) (72).

At 2022 ASCO Annual Meeting has been launched FIREFLY-1 (NCT04775485), a phase II study that will evaluate the safety and efficacy of tovorafenib monotherapy in pediatric and young adult patients with relapsed or progressive LGGs harboring a known activating BRAF alteration, such as BRAF V600 mutation, RAF gene fusions (BRAF and RAF1), including KIAA1549:BRAF fusions.

Tovorafenib is an oral small molecule, type II pan-RAF inhibitor that, in contrast to type I inhibitors, does not induce reactivation of the MAPK pathway (73). In the phase I study, tovorafenib has demonstrated clinical antitumor activity in 7/8 treated patients affected by LGG harboring RAF alterations, achieving 2 complete responses, 3 partial responses and 2 disease stabilizations.

Andrews et al. (74) recently published a systematic review and meta-analysis to assess the effectiveness of the use of BRAF inhibitors in V600-mutant gliomas. 127 studies were included in the meta-analysis: 66 case reports, 37 case series and 24 uncontrolled phase I/II trials. Across the included studies, data were available for 154 pediatric patients and 137 adult patients. Complete or partial responses were observed in 44% of pediatric and 54% of adult patients with LGG, and 56% of pediatric and 38% of adult patients with HGG. As expected, both PFS and OS was resulted longer in LGG patients than in HGG patients (median PFS resulted 13.0 versus 3.5 months in pediatric patients, and 5.9 versus 3.0 months in adult patients. Median OS was 6.1 versus 3 months in pediatric patients, and 9.5 versus 6.8 months for adults). No significant difference in OS were found between pediatric or adult patients who had anti-BRAF monotherapy compared to dual inhibition therapy. However, median PFS differed significantly between LGG and HGG patients treated with dual therapy (8.5 versus 2.9 months; p< 0.005).

Important insights can be drawn by analyzing clinical trials and meta-analysis: first, BRAF inhibitors, potentially in combination with MEK-inhibitors, might be a valuable therapeutic option for the treatment of recurrent BRAF mutated high grade gliomas and melanoma brain metastases, thus we can argue that these drugs appear to effectively penetrate the blood-brain-barrier. Notably, several patients also had leptomeningeal disease but reported radiographic responses (60), further demonstrating that this class of inhibitors adequately spread through brain tissue. Second, many of the reports describe rapid clinical improvement after initiating BRAF targeted therapy (48, 57, 60, 75). This point is crucial, considering that many of the patients described had a poor performance status: this class of drugs, despite the presence of the blood-brain-barrier, elicits an objective response in a short time, rapidly improving the symptoms of patients, similarly to what is observed in melanoma.

Although the dual BRAF/MEK inhibition may not provide any additional survival advantage compared with anti-BRAF agents alone, as suggested by the meta-analysis by Andrews et al. (74), the decreased toxicity and the possibility of reduced resistance with prolonged disease control would favor the combination of BRAF/MEK inhibitors over single-agents.

Another crucial point regards the appropriate timing for BRAF-targeted therapy initiation in gliomas, which is still unclear. In HGG, current clinical practice foresees starting anti-BRAF therapy only after the failure of standard therapy, at the recurrence of the disease.

In LGG, the timing for initiating anti-BRAF therapy is even more difficult to establish. It would be important to explore the possibility of anticipating this type of treatment in order to postpone radiotherapy as much as possible, given its high toxicity.

In addition to verifying the effectiveness of BRAF targeted therapy in HGG, it is essential to know the mechanisms of resistance to this class of drugs, mostly unclear, possibly by pursuing new tissue biopsy at the time of resistance. Future studies will investigate if the resistance mechanisms are different in LGG versus HGG and if they develop only in case of BRAF inhibitor monotherapy or even in case of dual BRAF/MEK inhibition (48).

EORTC Brain Tumor Group has recently launched a new and innovative study, EORTC-2013-BTG (NCT05259605), designed to create a large European database to register patients with rare primary brain tumors, including those recently redefined in the 2021 WHO classification, to provide updated disease profiles including data on age at diagnosis, clinical and imaging presentation, currently available treatments and outcomes, and molecular landscape. It is a combined retrospective and prospective registry study, where detailed biomarker research is an intrinsic and essential component of the project and where treatments are at the discretion of the local treating physician. This study will also analyze cohorts of BRAF mutant tumors and will provide molecular data for each selected tumor entity and a large amount of information on clinical features, treatment history and outcomes.

The rarity of BRAF mutations in glioma patients rises some issues related to financial investments and clinical trial design on patients with glioma. First the BRAF status assessment could be expansive and time consuming. However, trials investigating BRAF/MEK inhibitors in glioma allows the use of IHC and PCR testing especially for identification of BRAF^V600E^ mutation (59, 67). This could significantly reduce time and costs for BRAF assessment. Advanced techniques of next-generation sequencing could be adopted to identify rare BRAF mutations (59, 67)

Second, the rarity of BRAF-mutated glioma could be associated with long-term recruitment within clinical trials. The use of adaptive studies with Bayesian design can be a valid option to reduce the effective number of patients needed to demonstrate or refute BRAF inhibitor clinical activity (76, 77).

## Side effects of BRAF/MEK targeted therapy

Drug-class toxicities of BRAF inhibitors encompass pyrexia, arthralgia, fatigue, headache, cutaneous toxicities and growth of secondary skin neoplasms like cutaneous squamous-cell carcinoma palmoplantar erythrodysesthesia gastrointestinal side effects and elevated serum transaminases (**Table 3**) (78, 79).

**Table 3:** Treatment adverse events reported in VE-BASKET, ROAR and COMBI-AD trial.

VE-BASKET TRIAL (vemurafenib)			NCT01227889 (dabrafenib)			ROAR TRIAL (dabrafenib + trametinib)			COMBI-AD TRIAL (dabrafenib + trametinib)		
ADVERSE EVENT	ALL GRADES	GRADE 3-4	ADVERSE EVENT	ALL GRADES	GRADE 3-4	ADVERSE EVENT	ALL GRADES	GRADE 3-4	ADVERSE EVENT	ALL GRADES	GRADE 3-4
arthralgia	67%	/	pyrexia	8%	3%	fatigue	41%	41%	pyrexia	63%	5%
palmar-plantar erythrodysesthesia	38%	/	arthralgia	19%	1%	headache	38%	38%	fatigue	47%	4%
photosensitivity reaction	38%	/	fatigue	18%	1%	nausea	32%	32%	nausea	40%	1%
fatigue	29%	4%	squamous carcinoma keratoacanthoma	12%	4%	pyrexia	31%	31%	headache	39%	1%
pruritus	29%		palmar-plantar hyperkeratosis	16%	2%	neutropenia	10%	10%	chills	37%	1%
rash maculopapular	29%	13%	hyperkeratosis	12%	<1%	anaemia	22%	22%	diarrhea	33%	1%
folliculitis	25%	/	keratoacanthoma	12%	4%	AST/ALT elevation	17%	17%	vomiting	28%	1%
hyperkeratosis	25%	/	headache	5%	/	chills	14%	14%	arthralgia	28%	1%
keratosis pilaris	25%	/	nausea	1%	1%	/	12%	12%	rash	24%	/
headache	25%	/	vomiting	1%	/	ejection fraction decrease	10%	10%	cough	17%	/
diarrhoea	21%	/	neutropenia	1%	1%	dermatitis acneiform	10%	10%	hypertension	11%	6%
nausea	21%	/	diarrhoea	/	/	hypertension	3%	3%	elevated ATS/ALT	15%	4%
decreased appetite	21%	/	cardiac	/	/	diarrhoea	14%	14%	dermatitis acneiform	12%	/

For dabrafenib the most common side effects are cutaneous (hyperkeratosis, squamous cell carcinoma/keratoacanthoma, palmar-plantar erythrodysesthesia), pyrexia, fatigue, headache, and arthralgia, with toxicities of grade 3–4 very uncommon (**Table 3**) (78, 80)

For vemurafenib the most common toxicities are arthralgia, palmar–plantar dysesthesia, photosensitivity, fatigue, rash and cutaneous effects (as folliculitis, pruritus, hyperkeratosis) and gastrointestinal disorders (diarrhoea, nausea, decreased appetite) (**Table 3**) (78, 79).

The photosensitivity induced by vemurafenib is considered to be a property of the chemical structure of the drug, not related to its BRAF-inhibiting activity (78).

Most common treatment-related side effects of encorafenib include myalgia, palmoplantar erythrodysesthesia, nausea, arthralgia, alopecia and hyperkeratosis. Transient facial paresis has been reported in 8% of patients treated with encorafenib, and represents a specific toxicity for this drug, never reported in other BRAF inhibitors (78, 81).

MEK inhibitors class-effects include fatigue, anemia, acneiform dermatitis, pruritus, skin rash, gastrointestinal toxicity (nausea, diarrhea, vomiting), transaminases elevation, ocular toxicity (blurred vision, serous retinal detachment, retinal vein occlusion, chorioretinopathy), muscular problems, increased creatinine phosphokinase (CPK) and cardiovascular adverse events (hypertension and ventricular ejection fraction decrease), most of which occur early in treatment and decrease over time (78, 79).

Dabrafenib plus trametinib is the most common RAF plus MEK inhibitor regimen used in brain tumor patients, likely because of its relatively good efficacy in melanoma brain metastases (79, 82).

Overall, the use of the dabrafenib + trametinib combination, compared to monotherapies with BRAF inhibitors, results in a higher incidence of fever (~ 50%), dermatological events (~ 30%), gastrointestinal events (~30%), headache (~30%), ocular events, hyperglycaemia, cardiovascular events (~5%), pulmonary events, AST/ALT elevation (~15%), renal disorders (~1%) (78).

In the COMBI-AD trial evaluating the effectiveness of dabrafenib + trametinib as adjuvant treatment in melanoma patients, the following toxicities were observed in more than 20% of patients: pyrexia (63%), fatigue (47%), nausea (40%), headache (39%), chills (37%), diarrhea (33%), vomiting (28%), arthralgia (28%), and skin rash (24%) (79, 83). The most common grade 3 or 4 toxicities of combination therapy included pyrexia (5%), fatigue (4%), elevated ALT (4%) and AST (4%), and hypertension (6%). Recommended monitoring for dabrafenib and trametinib combination includes the following: dermatologic evaluation, cardiac function tests, hepatic function tests, retinal evaluation, as well as routine blood pressure evaluation, complete blood count, and serum glucose (79). Additionally, physicians should evaluate patients for signs and symptoms of skin toxicities and secondary infections, uveitis, bleeding, hemolytic anemia, and lung toxicity.

Notably, many toxicities as well as cutaneous toxicities, growth of secondary skin neoplasms or papulopustular rash may be significantly decreased and/or better tolerated in anti-BRAF/MEK combination regimens (79).

Toxicity profile of BRAF + MEK inhibitor combinations include (**Table 3**):

- dermatological events: rash, itching, dry skin, hair loss, photosensitivity reaction, keratinocytic proliferation and panniculitis, maculopapular exanthema, papulopustular exanthema or even eczema (79);
- gastrointestinal events: nausea, vomiting and diarrhea can be associated with abdominal pain and bleeding. Diarrhea has been reported up to 33%, resulting more frequent in the combination of BRAF + MEK inhibitor than in monotherapy (79, 84);
- musculoskeletal events such as arthralgia and myalgia. Arthralgia is very common with BRAF inhibitors as monotherapy but is less frequent for the combination strategies (79);
- cardiovascular events: are generally reversible, mild to moderate. QT prolongation is the most common cardiovascular event for BRAF inhibitors, whereas, decreased ejection fraction and left ventricular dysfunction have been described with MEK inhibitors. Fatal events due to arrhythmias or sudden cardiac death are very rare (85);
- ocular events: bilateral and multifocal macular oedema or retinal pigment epithelial detachment are common effects of anti-MEK therapy, occurring early within hours or days form therapy initiation (79, 86). Ocular toxicity is often asymptomatic and is diagnosed by optical coherence tomography;
- pulmonary events: interstitial pneumonitis is rare, occurring in up to 2% of the cases and is mainly caused by MEK inhibitors.

In general, BRAF plus MEK inhibitor therapy might be considered a safe therapy, if toxicity is monitored adequately by physicians that are expert with the clinical management of these drugs.

## Molecular mechanisms associated to target therapy resistance

Due to the only recent inclusion of RAF/MEK inhibitors in clinical trials for gliomas and central nervous system tumors, the knowledge toward resistance mechanisms to target therapy is limited and is mainly obtained by data coming from melanoma and colorectal cancer (69, 87–91). To date, the only study carried out on glioma patients confirmed that the acquisition of alternative BRAF mutations is a strategy adopted by tumors to overcome BRAF inhibition (91). Despite initial survival benefit, approximately half of all patients treated with anti-BRAF agents exhibits disease progression within 6–9 months after starting treatment: mechanisms of resistance to BRAF inhibitors are better known in melanoma and colorectal cancer and include genetic and epigenetic events leading to intrinsic primary or acquired secondary resistance (92–95).

Tumor heterogeneity and reactivation of the MAPK pathway are the two main mechanisms responsible of resistance in melanoma patients (50, 96).

Primary early resistance is rare in melanoma, occurring in 10–20% of cases, and is often associated with loss of PTEN, loss of NF1 or mutations in PI3K or AKT. The inactivation of PTEN is associated with scarce response rates to BRAF inhibitors (96–99). The combination of BRAF and PI3K inhibitors has been proposed as a possible strategy for overcoming this resistance and restoring apoptosis in deleted PTEN cells. Similarly, loss of NF1, a tumor suppressor of RAS, is responsible of the activation of the MAPK pathway. In this context, the dual combination of MEK and mTOR inhibitors seems to be a valuable strategy for overcoming resistance (100).

Secondary acquired resistance mechanisms are mainly associated with the reactivation of the MAP kinase pathway through several mechanisms: BRAF amplification and alternative splicing of BRAF, increased expression of receptor tyrosine kinases (particularly platelet derived grow factor receptor beta and insulin-like grow factor I receptor), hotspot activating mutations in NRAS, KRAS, PI3K, MEK, with reactivation of PI3K-PTEN-mTOR axis. Secondary BRAF mutations have not been described as genetic events drivers of secondary acquired resistance (96, 98, 101, 102).

A study carried out by Schreck KC et al. identified several mechanisms leading to BRAF resistance including for example EGFR hyperexpression, and mutations involving ERRFI1, BAP1, ANKHD1, and Map2k1 (103).

A study showed that BRAFV600E inhibitor resistant cells upregulate pro-survival mediators such as Wnt, and additionally increase receptor tyrosine kinase activity, including EGFR and Axl, promoting resistance to BRAFV600E inhibition. Given that, in BRAF mutated colon cancer, and glioma the combination of BRAF inhibitors with anti-EGFR agents is a promising strategy (104).

In BRAF V600E mutated colon cancer, the use of targeted single agents has shown limited benefit (105, 106). A possible reason, hypothesized and sustained by data derived from preclinical studies, is that BRAF V600E inhibition by an anti-BRAF agent causes a rapid feedback phenomenon of EGFR activation which continues to “fuel” cell proliferation (90, 105). In melanoma cells, instead, characterized by low levels of EGFR expression, this feedback phenomenon has not been found. Given that, in BRAF mutated colon cancer, the combination of BRAF inhibitors with anti-EGFR agents is a promising strategy. The PLK1 activity controls a polarity checkpoint and compensates for BRAF/MAPK inhibition in CD133(+) tumor cells, suggesting the need for concurrent PLK1 inhibition to improve antitumor activity against a therapy-resistant cell compartment (69).

## Conclusion and future perspectives

Current data suggest that BRAF V600E is therapeutically actionable in gliomas. Nevertheless, the rarity of BRAF V600E mutation in adult CNS tumors in general and in HGG in particular has limited the conduction of prospective trials specifically designed for this population, and patients with brain tumors have been usually enrolled in little cohorts of “basket” trials including a variety of histologic subtypes, making it challenging to achieve certain, reproducible and generalizable results.

Although BRAF V600E mutation is uncommon in adult HGG patients, considering the very limited treatment options currently available for this population, the screening for BRAF point mutations would increase in the next years.

Further prospective studies are needed to verify the efficacy of BRAF targeted therapy in this population and intense research should be devoted to the study of resistance mechanisms, till now largely unknown. Given high response rate and significant clinical benefit, poor performance status and leptomeningeal disease should not be considered exclusion criteria when designing new trials in this population. Future research should also determine the optimal timing for starting BRAF/MEK inhibitors and should improve the management of toxicities and the quality of life by introducing patient-reported outcomes in clinical trials. If the results of phase II trials such as NCT02684058 and ROAR will be confirmed in larger clinical studies the treatment algorithm of BRAF mutated glioma could be upset with BRAF/MEK inhibitors adopted as upfront or early systemic treatment.

Collaborative efforts, such as the EORTC-2013-BTG trial, aimed at collecting data on these rare patients, will be crucial for future research.

## Author contributions

All authors contributed to the article and approved the submitted version.
